# Effect of care environment on educational attainment among orphaned and separated children and adolescents in Western Kenya

**DOI:** 10.1186/s12889-022-12521-5

**Published:** 2022-01-18

**Authors:** Dorothy Apedaile, Allison DeLong, Edwin Sang, David Ayuku, Lukoye Atwoli, Omar Galárraga, Paula Braitstein

**Affiliations:** 1 Division of Epidemiology, Dalla Lana School of Public Health, University of Toronto, 155 College Street, Toronto, ON M5T 3M7 Canada; 2 Department of Biostatistics, School of Public Health, Brown University, Providence, RI USA; 3 Academic Model Providing Access to Healthcare (AMPATH), Eldoret, Kenya; 4 Department of Mental Health and Behavioural Sciences, College of Health Sciences, School of Medicine, Moi University, Eldoret, Kenya; 5 Aga Khan University Medical College, East Africa, Nairobi, Kenya; 6 Department of Health Policy, Services, and Practice, School of Public Health, Brown University, Providence, RI USA

**Keywords:** Education, Orphans, Children’s rights, Kenya, Residential care, Institutions, Foster care, Family-based care

## Abstract

**Background:**

There are approximately 140 million orphaned and separated children (OSCA) around the world. In Kenya, many of these children live with extended family while others live in institutions. Despite evidence that orphans are less likely to be enrolled in school than non-orphans, there is little evidence regarding the role of care environment. This evidence is vital for designing programs and policies that promote access to education for orphans, which is not only their human right but also an important social determinant of health. The purpose of this study was to compare educational attainment among OSCA living in Charitable Children’s Institutions and family-based settings in Uasin Gishu County, Kenya.

**Methods:**

This study analyses follow up data from a cohort of OSCA living in 300 randomly selected households and 17 institutions. We used Poisson regression to estimate the effect of care environment on primary school completion among participants age ≥ 14 as well as full and partial secondary school completion among participants age ≥ 18. Risk ratios and 95% confidence intervals were estimated using a bootstrap method with 1000 replications.

**Results:**

The analysis included 1406 participants (495 from institutions, 911 from family-based settings). At baseline, 50% were female, the average age was 9.5 years, 54% were double orphans, and 3% were HIV-positive. At follow-up, 76% of participants age ≥ 14 had completed primary school and 32% of participants age ≥ 18 had completed secondary school. Children living in institutions were significantly more likely to complete primary school (aRR: 1.18, 95% CI: 1.10–1.28) and at least 1 year of secondary school (aRR: 1.28, 95% CI: 1.18–1.39) than children in family-based settings. Children living in institutions were less likely to have completed all 4 years secondary school (aRR: 0.79, 95% CI: 0.43–1.18) than children in family-based settings.

**Conclusion:**

Children living in institutional environments were more likely to complete primary school and some secondary school than children living in family-based care. Further support is needed for all orphans to improve primary and secondary school completion. Policies that require orphans to leave institution environments upon their eighteenth birthday may be preventing these youth from completing secondary school.

## Introduction

Education is widely recognized as a fundamental social determinant of health [1–3]. Education is also recognized as a human right under Article 28 of the UN Convention on the Rights of the Child, which states that primary education should be free and compulsory and that secondary education should be available and accessible to all children [4]. Increased educational attainment is associated with positive health outcomes, higher socioeconomic status, greater access to healthcare, increased life expectancy, and improvements in childhood mortality [5–10].

Education has been identified as a global priority in both the Millennium Development Goals and the Sustainable Development Goals [11]. However, there continue to be disparities in educational attainment both between countries and within specific population groups. While net primary school enrolment around the world increased to 91% in 2015 from 83% in 2000, children in the poorest households were four times less likely to attend school than children in the richest households in developing countries [12]. Sub-Saharan Africa saw the greatest increases in primary school enrolment during the Millennium Development Goals era, but continued to have the lowest literacy rates among youth compared to other regions [12].

Orphaned and separated children and youth (OSCA) have particularly low educational attainment [13, 14]. UNICEF defines an orphan as a child under 18 years of age who has lost one or both parents to any cause of death [15]. Based on UNICEF’s definition, there were an estimated 140 million orphans in the world in 2015, of whom 15.1 million had lost both parents [15]. Orphans are less likely to be attending school; more likely to have lower academic achievement and higher absenteeism; and are less likely to be in the right grade level for their age than non-orphans [16–18]. Previous research has found that there are many barriers to educational attainment for orphans, including the psychological trauma (i.e. grief and depression) and material impact (i.e. lack of money for school fees) of losing a parent [17], missing time from school to care for a sick parent before they die [17], food insecurity (i.e. a child may go to the street in search of food rather than going hungry all day at school) [14, 19], and the cost of school fees and ancillary costs (e.g. uniform, school shoes, school bag) [14]. Among OSCA who did not complete their educations, leaving school early has been identified as a source of significant psychological distress [19].

According to UNICEF, 52 million of the world’s orphans are living in Africa [15]. Globally, an estimated 16.6 million children have lost both parents to HIV/AIDS, of whom 90% live in sub-Saharan Africa [20]. In 2012, there were an estimated 1.8 million orphans living in Kenya, of whom 15% were double orphans who had lost both their parents [21]. The majority of OSCA live with their surviving parent or extended family [15]. However, households caring for OSCA in Kenya are often extremely poor and many are unable to meet the basic needs of the children in their care [13]. The large number of children in need of care has resulted in many children living in institutional settings (e.g., orphanages), while research from a range of countries has demonstrated negative short and long-term physical and mental health outcomes for children growing up in institutional care environments [22, 23].

However, little is known about the impact of different care environments on educational attainment, despite considerable attention to both the lack of education among OSCA as well as the negative impacts of institutions [24]. Understanding the impact of institutions and other models of care on educational attainment is vital to designing programs and policies that promote access to education for OSCA. The positive, long-term health and social impacts of increased education are particularly important OSCA, who already face discrimination and less family support [16, 25]. The objective of this analysis was to investigate the impact of care environment on primary and secondary school completion among OSCA living in western Kenya, a country that has affirmed the right to education and the right of children to free and compulsory basic education in the constitution [26]. Due to the negative impacts of institutions in other areas of health and wellbeing, our hypothesis was that children living in institutions would complete less education than children living in family-based settings.

## Methods

### Study design

The Orphaned and Separated Children’s Assessments Related to their (OSCAR’s) Health and Well-Being Project is a two-phase longitudinal cohort study investigating the effects of care environment on the physical and psychosocial well-being of OSCA in Uasin Gishu County, Kenya [27]. The study enrolled participants < 18 years of age from May 31, 2010 to April 24, 2013. Phase 1 ran from 2010 to 2015 and Phase 2 ran from 2016 to 2019. The OSCAR cohort comprises participants from 300 randomly selected households caring for OSCA and 19 Charitable Children’s Institutions (CCIs) (of 21 in the county at the time of study start-up). All children, both orphaned and non-orphaned, from each household or institution were eligible to participate in the study. Among the 300 households, recruitment was designed to include 100 households receiving the government cash transfer for the support of orphaned children, 100 households not receiving the cash transfer from within the same sub-Locations as those receiving the cash transfers, and 100 households not receiving the cash transfer from different sub-Locations than those receiving the cash transfers. Sub-Locations are administrative boundaries within Uasin Gishu County, each headed by an Assistant Chief [27]. In-depth details about the OSCAR cohort’s study design, setting, and recruitment have been previously reported [27].

### Study population

This report includes all participants who were orphaned or separated at the time of enrolment into Phase 1. Separated children were defined as those whose biological mother or father was potentially alive, but functionally not part of the child’s life. Since questions regarding education were introduced in Phase 2, the sample is further restricted to participants who completed at least one Phase 2 visit. Of the 19 CCIs recruiting in Phase 1, two were not eligible to participate in Phase 2 since they provided shorter-term care. Therefore, participants from these two CCIs did not enrol in Phase 2 of the OSCAR study and were not included in this analysis.

### Human subjects’ protections

The Moi University College of Health Sciences and Moi Teaching and Referral Hospital Institutional Research and Ethics Committee, the Indiana University Institutional Review Board, and the University of Toronto Research Ethics Boards approved this study. This study conforms to the principles embodied in the Declaration of Helsinki. Written informed consent for participation was provided by the head of household or Director of the CCI. Individual written informed assent was provided by each child aged 7 years and above. Fingerprints were used for both children and guardians who were unable to sign or write their name.

### Patient and public involvement in research

This study utilized community-based, participatory processes to inform the research questions, hypotheses, and methods, as detailed elsewhere [27]. To summarize briefly, the Children’s Officers in the region and representatives from CCIs were initially consulted prior to the funding application. They were requested to provide input as to whether such a study would be important from their perspective, and what their priority questions and concerns were. In addition, traditional community assemblies were held in some of the target communities to identify community concerns and priorities with respect to the care of orphaned and vulnerable children. These assemblies were also held following the initiation of the study to maintain regular contact with the community and disseminate findings. We formed an Advisory Board early on, consisting of representatives from communities, CCIs, and Children’s Officers, and this board met regularly throughout the life of the study. Our study disseminated findings through the monthly Uasin Gishu Children’s Services Forum, through additional traditional community assemblies, and through the study website [28].

### Procedures

Data collection was conducted in situ at CCIs and at the OSCAR Project clinic for participants from households. Annually, participants completed a standardized clinical encounter and those ≥10 years of age also completed a psychosocial encounter. The clinical encounter was an enhanced well-child ‘check-up’ administered by the project medical officer (i.e., physician) that included a complete physical history and review of health symptoms. A psychosocial encounter measured education and employment, material well-being, behaviours and risks, peer and family relationships, and mental health. The psychosocial assessment was self-administered for those who could read and write or psychologist-administered for those that could not adequately read or write. A clinical psychologist was always available during the assessments to assist in case of questions, lack of understanding, or distress. Follow-up of cases requiring individual counselling or case management took place on a case by case basis as needed, by study staff. Household level data, including age and education level of the household head, and other characteristics of the care environments (such as shelter type and source of water) were obtained through annual site assessments administered by the Project Manager for CCIs, and Community Health Workers in the participating households.

### Independent variables

The primary exposure of interest was care environment (institutional or family-based) upon study enrolment [13]. Sociodemographic characteristics were ascertained at the baseline clinical encounter, including age, sex, orphan/separated status (maternal, paternal, or both), HIV status (positive, negative, unknown), hospitalizations in the past year, area of residence (rural or urban) and time living with caregiver (< 6 months, 6 months-2 years, 2–5 years, > 5 years, all the child’s life). The guardian’s level of education at enrolment (none, primary, secondary, vocational, university) was assessed through a site assessment. Follow-up time was defined as the time between the first and last encounters each individual participated in.

### Educational outcomes

The Kenyan education system includes 8 years of primary school from ages 6 to 13 (Class 1 to Class 8) and 4 years of secondary school from age 14 to 17 (Form 1 to Form 4). Participants were asked to identify the highest class they had completed in school, if they had ever attended school, if they were currently attending school, and how many days of school they had missed in the past 4 weeks (none, 1–2 days, 3–5 days, > 5 days). The primary outcomes were completion of primary school (Class 8 or higher among participants age 14 or older), completion of one or more years of secondary school (Form 1 or higher among participants age 18 or older), and completion of all 4 years of secondary school (Form 1 or higher among participants age 18 or older) at the time of the participant’s last follow up visit.

### Statistical analysis

Descriptive statistics at baseline were calculated for both the initial study population and the population with at least one Phase 2 visit, overall and by care environment. Mean values and standard deviations are reported for normally distributed continuous characteristics, median values and interquartile ranges are reported for non-normally distributed continuous characteristics, and frequencies and percentages are reported for categorical characteristics. Differences in baseline characteristics by care environment were assessed using Pearson’s Chi-Squared tests for categorical characteristics and two-sample t-tests for continuous characteristics. To assess loss to follow up, Pearson’s Chi-Squared tests were used to compare categorical characteristics of participants who completed a Phase 2 visit to participants who did not complete a Phase 2 visit. Continuous characteristics were compared using a two-sample t-test. Educational outcomes at the last follow-up visit were described by frequency and percentage for each care environment.

The effect of care environment on each educational outcome (primary school completion, partial secondary school completion, and secondary school completion) was estimated separately using bootstrapped Poisson regression. Poisson regression was chosen to present a risk ratio, the ratio of the cumulative incidence of school completion in the exposed (children from CCIs) and unexposed (children from FBS) groups. Results are reported unadjusted and adjusted for sex, orphan status at enrolment, HIV status at enrolment, and hospitalization in the past year. A sensitivity analysis was conducted to adjust for area (urban or rural).

The risk ratios and 95% confidence intervals were estimated using bootstrap resampling with 1000 replications. Sampling of participants with replacement was conducted within each original sampling stratum (CCI, non-cash transfer household, same sub-Location household, and different sub-Location household) to account for clustering. The regression models were fit using inverse probability-of-censoring weights to reduce selection bias from the differential loss to follow by simulating a pseudo-population where the loss to follow up was random [29]. These weights estimate the probability of each participant completing a Phase 2 visit based on their characteristics. The weights were calculated using generalized additive models (GAM) stratified by CCI, non-cash transfer household, same sub-Location household, and different sub-Location household. The GAMs predicted the probability of a participant completing a Phase 2 visit using a smoothed function on age at enrolment and adjusted for sex, area (urban or rural), orphan status at baseline, time with guardian at baseline, recent hospitalization at baseline, and HIV status at baseline.

## Results

### Characteristics of participants

Table 1 describes the baseline characteristics of the 2099 participants enrolled in the OSCAR study who were eligible to participate in Phase 2 (i.e. excluding participants from the 2 CCIs that were not invited to participate in Phase 2). At baseline, 45% (*n* = 939) of participants were living in a CCI while 55% (*n* = 1160) of participants were living in a family-based setting (FBS). Among the participants living in a FBS, 36% (*n* = 738) were living in households that received the government cash transfer (data not shown). The mean age was 10.3 years,49% of participants were female, and there were 65 participants (3%) who were HIV-positive at baseline.

**Table 1:** Population characteristics at baseline among all OSCAR participants

	Total *N* = 2099 N (%)	CCI *N* = 939 N (%)	FBS *N* = 1160 N (%)	***P***-value
**Age [mean (sd)]**^**a**^	10.3 (4.7)	10.2 (4.65)	10.3 (4.74)	0.99
**Follow-up time [median (IQR)]**^**a**^	6.7 (3.0–7.5)	5.6 (2.2–7.7)	6.9 (5.5–7.5)	< 0.001
**Gender**				
Female	1035 (49.3)	434 (46.2)	601 (51.8)	0.01
Male	1064 (50.7)	505 (53.8)	559 (48.2)	
Missing	0 (0.0)	0 (0.0)	0 (0.0)	
**Area**				
Rural	1145 (54.5)	384 (40.9)	761 (65.6)	< 0.001
Urban	926 (44.1)	527 (56.2)	399 (34.4)	
Missing	28 (1.3)	28 (3.0)	0 (0.0)	
**Orphan Status**				
Double Orphan	1257 (59.9)	811 (86.4)	446 (38.4)	< 0.001
Maternal Orphan	187 (8.9)	66 (7.0)	121 (10.4)	
Paternal Orphan	655 (31.2)	62 (6.6)	593 (51.1)	
Missing	0 (0.0)	0 (0.0)	0 (0.0)	
**Time with guardian**				
< 6 months	106 (5.1)	93 (9.9)	13 (1.1)	< 0.001
6 months – 2 years	284 (13.5)	252 (26.8)	32 (2.8)	
2–5 years	457 (21.8)	314 (33.4)	143 (12.4)	
5+ years	351 (16.7)	234 (24.9)	117 (10.1)	
Whole life	875 (41.7)	27 (2.9)	848 (73.5)	
Missing	26 (1.2)	19 (2.0)	7 (0.6)	
**Guardian education level**				
None	233 (11.0)	0 (0.0)	233 (20.2)	< 0.001
Primary	627 (29.9)	1 (0.1)	626 (54.2)	
Secondary	375 (17.9)	133 (14.2)	242 (21.0)	
Vocational	563 (26.8)	560 (59.6)	3 (0.3)	
University	245 (11.7)	245 (26.1)	0 (0.0)	
Other	51 (2.4)	0 (0.0)	41 (4.4)	
Missing	5 (0.2)	0 (0.0)	5 (0.4)	
**Hospitalized in past year**				
Yes	84 (4.1)	55 (5.9)	29 (2.5)	< 0.001
No	1949 (92.9)	833 (88.7)	1116 (97.5)	
Missing	67 (3.2)	51 (5.4)	15 (1.3)	
**HIV Status at enrollment**				
HIV negative	2032 (96.8)	895 (95.3)	1137 (98.0)	0.001
HIV positive	65 (3.1)	42 (4.5)	23 (2.0)	
Missing	2 (0.1)	2 (0.2)	0 (0.0)	

^**a**^reported in years

*Abbreviations*: *CCI* Charitable Children’s Institution, *FBS* Family-Based Setting, *HIV* Human immunodeficiency virus

Among participants who completed at least one Phase 2 visit, the mean age of participants from CCIs at their last Phase 2 visit was 15.5 years, while participants from FBS had a mean age of 17.3 years at their last Phase 2 visit (Table 2). Among OSCA in FBS, the majority (65%) were living in rural areas, while 48% of OSCA in a CCI were living in rural areas. At baseline, 762 (54%) participants were double orphans, with most double orphans living in a CCI. In FBS, 76% of OSCA had lived with their guardian for their whole life at baseline, while 75% of OSCA from CCIs had lived with their guardian for less than 5 years at baseline. Most guardians in a CCI had vocational (47%) or university education (36.2%), while most guardians in a FBS had either no education (20%) or only primary education (55%).

**Table 2:** Population characteristics among participants with at least one Phase 2 visit

	Total *N* = 1406 N (%)	CCI *N* = 495 N (%)	FBS *N* = 911 N (%)	***P***-value
**Age at baseline [mean (sd)]**^**a**^	9.5 (4.6)	8.3 (4.1)	10.2 (4.7)	< 0.001
**Age at last visit [mean (sd)]**^**a**^	16.6 (4.5)	15.5 (3.9)	17.3 (4.7)	< 0.001
**Follow-up time [median (IQR)]**^**a**^	7.4 (6.7–7.8)	7.9 (6.5–8.0)	7.3 (6.7–7.6)	< 0.001
**Gender**				
Female	708 (50.4)	236 (47.7)	472 (51.8)	0.14
Male	698 (49.6)	259 (52.3)	439 (48.2)	
Missing	0 (0.0)	0 (0.0)	0 (0.0)	
**Area**				
Rural	833 (59.2)	237 (47.9)	596 (65.4)	< 0.001
Urban	545 (38.8)	230 (46.5)	315 (34.6)	
Missing	28 (2.0)	28 (5.7)	0 (0.0)	
**Orphan Status at baseline**				
Double Orphan	762 (54.2)	434 (87.7)	328 (36.0)	< 0.001
Maternal Orphan	131 (9.3)	35 (7.1)	96 (10.5)	
Paternal Orphan	513 (36.5)	26 (5.3)	487 (53.5)	
Missing	0 (0.0)	0 (0.0)	0 (0.0)	
**Time with guardian at baseline**				
< 6 months	56 (4.0)	47 (9.5)	9 (1.0)	< 0.001
6 months – 2 years	170 (12.1)	152 (30.7)	18 (2.0)	
2–5 years	271 (19.3)	170 (34.3)	101 (11.1)	
5+ years	176 (12.5)	92 (18.6)	84 (9.2)	
Whole life	712 (50.6)	18 (3.6)	694 (76.2)	
Missing	21 (1.5)	16 (3.2)	5 (0.6)	
**Guardian education level**				
None	181 (12.9)	0 (0.0)	181 (19.9)	< 0.001
Primary	501 (35.6)	1 (0.2)	500 (54.9)	
Secondary	268 (19.1)	84 (17.0)	184 (20.2)	
Vocational	234 (16.6)	231 (46.7)	3 (0.3)	
University	179 (12.7)	179 (36.2)	0 (0.0)	
Other	39 (2.8)	0 (0.0)	39 (4.3)	
Missing	4 (0.3)	0 (0.0)	4 (0.4)	
**Hospitalized in past year**				
Yes	31 (2.2)	10 (2.0)	21 (2.3)	0.71
No	1356 (96.4)	481 (97.2)	875 (96.0)	
Missing	19 (1.4)	4 (0.8)	15 (1.7)	
**HIV Status at enrollment**				
HIV negative	1359 (96.7)	463 (93.5)	896 (98.4)	< 0.001
HIV positive	46 (3.3)	31 (6.3)	15 (1.6)	
Missing	1 (0.1)	1 (0.2)	0 (0.0)	

^**a**^reported in years

*Abbreviations*: *CCI* Charitable Children’s Institution, *FBS* Family-Based Setting, *HIV* Human immunodeficiency virus

Table 3 compares participants who completed a Phase 2 visit to participants who did not complete a Phase 2 visit. OSCA from FBS were significantly more likely to have completed a Phase 2 visit than OSCA from CCIs (*P* <  0.001). Participants who were younger when they enrolled in the study, living in rural areas, had at least one parent, and had lived with their guardians for longer were more likely to participate in Phase 2 (*P* <  0.001). HIV status at baseline and gender were not significantly associated with Phase 2 participation.

**Table 3:** Comparison of participants with at least one Phase 2 visit to participants that did not participate in Phase 2

	Participants without a Phase 2 Visit *N* = 693 N (%)	Participants with a Phase 2 Visit *N* = 1406 N (%)	***p***-value
**Care environment**			
Charitable Children’s Institution	444 (64.1)	495 (35.2)	< 0.001
Family-based setting	249 (35.9%)	911 (64.8)	
**Age at baseline [mean (sd)]**^**a**^	11.7 (4.6)	9.5 (4.6)	< 0.001
**Age at last visit [mean (sd)]**^**a**^	13.7 (4.8)	16.7 (4.5)	< 0.001
**Gender**			
Female	327 (47.2)	708 (50.4)	0.17
Male	366 (52.8)	698 (49.6)	
Missing	0 (0.0)	0 (0.0)	
**Area**			
Rural	312 (45.0)	833 (59.2)	< 0.001
Urban	381 (55.0)	545 (38.8)	
Missing	0 (0.0)	28 (2.0)	
**Orphan status at baseline**			
Double Orphan	495 (71.4)	762 (54.2)	< 0.001
Maternal Orphan	56 (8.1)	131 (9.3)	
Paternal Orphan	142 (20.5)	513 (36.5)	
Missing	0 (0.0)	0 (0.0)	
**Time with guardian at baseline**			
< 6 months	50 (7.2)	56 (4.0)	< 0.001
6 months – 2 years	114 (16.5)	170 (12.1)	
2–5 years	186 (26.8)	271 (19.3)	
5+ years	175 (25.3)	176 (12.5)	
Whole life	163 (23.5)	712 (50.6)	
Missing	5 (0.7)	21 (1.5)	
**Guardian education level**			
None	52 (7.5)	181 (12.9)	< 0.001
Primary	126 (18.2)	501 (35.6)	
Secondary	107 (15.4)	268 (19.1)	
Vocational	329 (47.5)	234 (16.6)	
University	66 (9.5)	179 (12.7)	
Other	12 (1.7)	39 (2.8)	
Missing	1 (0.1)	4 (0.3)	
**HIV Status at baseline**			
HIV negative	673 (97.1)	1359 (96.7)	0.51
HIV positive	19 (2.7)	46 (3.3)	
Missing	1 (0.1)	1 (0.1)	

^a^ reported in years

*Abbreviations*: *HIV* Human immunodeficiency virus

### Educational outcomes

Table 4 presents educational characteristics at follow-up stratified by care environment and age group. All but 2 participants reported that they had previously attended school. Almost all participants aged 6–13 (99%) and 14–17 (96%) reported that they were currently attending school. Among participants over the age of 18, 94% of those from CCIs were currently attending school while 56% of those from FBS were currently attending school. Across all age groups and care environments, over 90% of participants currently attending school reported missing 2 or fewer days of school in the past 4 weeks.

**Table 4:** Educational participation in school-aged children and youth at last follow up visit

	CCI			FBS		
	Age 6–13 *N* = 172 N (%)	Age 14–17 *N* = 167 N (%)	Age 18+ *N* = 156 N (%)	Age 6–13 *N* = 220 N (%)	Age 14–17 *N* = 244 N (%)	Age 18+ *N* = 444 N (%)
**Ever attended school**						
*Yes*	172 (100.0)	167 (100.0)	156 (100.0)	218 (99.1)	244 (100.0)	444 (100.0)
*Missing*	0 (0.0)	0 (0.0)	0 (0.0)	0 (0.0)	0 (0.0)	0 (0.0)
**Currently attending school**						
*Yes*	171 (99.4)	166 (99.4)	146 (93.6)	216 (98.2)	230 (94.3)	247 (55.6)
*Missing*	0 (0.0)	0 (0.0)	0 (0.0)	2 (0.9)	0 (0.0)	0 (0.0)
**Days of school missed in past 4 weeks**^**a**^						
*None*	148 (86.5)	154 (92.8)	132 (90.4)	194 (89.8)	198 (86.1)	208 (84.2)
*1–2 days*	19 (11.1)	10 (6.0)	5 (3.4)	14 (6.5)	18 (7.8)	14 (5.7)
*3–5 days*	3 (1.7)	1 (0.6)	4 (2.7)	3 (1.4)	7 (3.0)	8 (3.2)
*> 5 days*	1 (0.6)	1 (0.6)	5 (3.4)	2 (0.9)	6 (2.6)	13 (5.2)
*Missing*	0 (0.0)	0 (0.0)	0 (0.0)	5 (2.3)	1 (0.4)	0 (0.0)
**Primary school**						
*None completed*	15 (8.7)	0 (0.0)	0 (0.0)	19 (8.6)	2 (0.8)	1 (0.2)
*Some completed*^b^	153 (88.9)	62 (37.1)	2 (1.3)	195 (88.6)	123 (50.4)	52 (11.7)
*All completed*^c^	2 (1.2)	105 (62.9)	154 (98.7)	2 (0.9)	118 (48.4)	391 (88.1)
*Missing*	2 (1.2)	0 (0.0)	0 (0.0)	4 (1.8)	1 (0.4)	0 (0.0)
**Secondary school**						
*None completed*	NA	97 (58.1)	8 (5.1)	NA	169 (69.3)	112 (25.2)
*Some completed*^d^	NA	70 (41.9)	118 (75.6)	NA	71 (29.1)	169 (38.1)
*All completed*^e^	NA	0 (0.0)	30 (19.2)	NA	3 (1.2)	163 (36.7)
*Missing*	NA	0 (0.0)	0 (0.0)	NA	1 (0.4)	0 (0.0)

^a^Among participants currently attending school

^b^Highest level completed is between Grade 1 and Grade 7

^c^Highest level completed is Grade 8 or higher

^d^Highest level completed is between Form 1 and Form 3

^e^Highest level completed is Form 4 or higher

*Abbreviations*: *CCI* Charitable Children’s Institution, *FBS* Family-Based Setting

For OSCA living in a CCI, 105 participants age 14–17 (63%) and 154 participants age 18 or older (99%) completed primary school. For OSCA living in a FBS, 118 participants age 14–17 (48%) and 391 participants age 18 or older (88%) completed primary school. Among participants age 18 or older from a CCI, 30 (19%) completed all 4 years of secondary school compared to 163 (37%) of those from FBS. However, 112 (25%) of participants age 18 or older from FBS had not completed any secondary school compared to 8 (5.1%) participants from a CCI.

The results of the Poisson regression with inverse probability weights are presented in Table 5. Area (rural/urban) did not change the main effect and was not included in the final model. Among participants aged 14 and over, OSCA living in a CCI were 1.18 times more likely to complete primary school (95% CI 1.10–1.28) than OSCA living in a FBS after adjusting for potential confounders. Among participants aged 18 and over, OSCA living in a CCI were also more likely to complete one or more years of secondary school compared to OSCA living in a FBS (aRR = 1.28, 95% CI 1.18–1.39). Although OSCA age 18+ in a CCI were 0.79 times as likely to complete all 4 years of secondary school when compared to those in a FBS (95% CI 0.43–1.18) after adjustment, this result was not statistically significant.

**Table 5:** Effect of Care Environment on Educational Attainment at last follow-up visit from Poisson regression analyses

Characteristic	Primary Completion^**a**^		Completed 4 Years of Secondary School^**b**^		Completed 1+ Years of Secondary School^**b**^	
	RR (95% CI)	aRR (95% CI)^**c**^	RR (95% CI)	aRR (95% CI)^**c**^	RR (95% CI)	aRR (95% CI)^**c**^
**Environment** (CCI vs FBS)	1.17 (1.10–1.24)	1.18 (1.10, 1.28)	0.86 (0.44, 1.37)	0.79 (0.43, 1.18)	1.29 (1.21, 1.36)	1.28 (1.18, 1.39)
**Sex** (Male vs Female)		0.90 (0.84, 0.96)		0.65 (0.46, 0.96)		0.93 (0.87, 0.99)
**Orphan status**						
Double orphan (ref)		1.00 (ref)		1.00 (ref)		1.00 (ref)
Maternal orphan		0.93 (0.80, 1.05)		0.85 (0.50, 1.28)		1.03 (0.90, 1.16)
Paternal orphan		1.04 (0.95, 1.12)		0.92 (0.69, 1.23)		1.01 (0.92, 1.11)
**HIV Positive**		0.94 (0.75, 1.10)		0.98 (0.32, 1.76)		1.03 (0.82, 1.20)
**Hospitalized in past year**		1.03 (0.84, 1.20)		0.72 (0.20, 1.38)		0.88 (0.59, 1.12)

^a^Among participants aged 14 or older

^b^Among participants aged 18 or older

^c^Adjusted for sex, orphan status, HIV status, and hospitalization in the past year

*Abbreviations*: *RR* Risk ratio, *CI* Confidence interval, *aRR* Adjusted risk ratio, *CCI* Charitable Children’s Institution, *FBS* Family-Based Setting

## Discussion

Our findings suggest that there is room for improvement in both primary and secondary school completion for OSCA in Uasin Gishu County, Kenya, regardless of care environment. While most school-aged OSCA were currently attending school, many had not achieved the educational milestones expected for their age. This was particularly pronounced in OSCA over the age of 18, of whom only a third had completed high school. OSCA living in a CCI were significantly more likely to complete primary school (among participants aged 14 and over) and at least 1 year of secondary school (among participants aged 18 and over) than those living in family-based care, after adjusting for potential confounding variables. There was no statistically significant effect of care environment on secondary school completion among participants aged 18 and over after adjustment.

Previous research has found that orphans are less likely to be in the right grade for their age than non-orphans [30]. While we did not compare orphans to non-orphans in this study, our results suggest that many OSCA are completing primary school later than expected, despite high attendance and low absenteeism. This somewhat contradicts other studies that found high absenteeism among orphans and lower school enrollment and attendance [14, 30, 31]. These differences may be explained by Kenya’s system of free primary education, which was re-introduced in 2003 during the era of the Millennium Development Goals [32]. However, there continue to be concerns regarding quality, class sizes, other fees, and physical capacity in schools [32]. There also continue to be financial barriers to attending secondary school, particularly for OSCA who often live in low-income households [14, 31]. Very few OSCA in our study completed all 4 years of secondary school and one fifth had completed no secondary school at all by the time they had turned 18.

Although institutions have been associated with a wide range of negative impacts on OSCA, some research has found that health, emotional, and cognitive functioning were no worse for children living in institutions compared to those living in the community [33, 34]. Consistent with these findings, we found that OSCA living in a CCI were more likely to complete primary school and one or more years of secondary school compared to children living in family-base care. Potential explanations for this include higher education levels among guardians in institutions compared to guardians in FBS and fewer financial barriers to attending school due to fees being covered by the CCI [35]. In addition, OSCA living in a CCI may have more time for school due to fewer responsibilities at home and less need to earn income [13].

Despite consistently higher primary school and partial secondary school completion, OSCA from CCIs were less likely to fully complete secondary school, though this result was not statistically significant. It is possible that their education is being interrupted due to “aging out” of care when they turn 18 and needing to find their own shelter and employment [36]. In contrast, OSCA from FBS may be able to stay in their households and continue attending school with the support of their extended family. While government policy requires that CCI’s develop exit plans for youth, the transition can be difficult and youth may lack the support needed to complete their studies [25]. In contrast, youth living in a FBS are not necessarily required to move out upon turning 18. This impact of “aging out” of care is not unique to Kenya’s CCI system. Youth aging out of state foster care in Canada have low rates of academic achievement and report a sense of anxiety and abandonment [37, 38]. In the United States, one quarter of former foster youth age 23–24 did not have a high school diploma. Youth who remained in care after the age of 18 reported better educational and employment outcomes [39].

This study has several strengths. The sampling design allows us to compare educational outcomes for OSCA living in institutions to those of OSCA living in family-based settings within the same geographical region. Detailed information was collected on study participants, allowing us to adjust for many important confounding variables. This study also has some limitations. Educational outcomes were self-reported by participants and are subject to recall bias and reporting bias. We were also unable to measure education quality or achievement. In addition, follow up time was shorter for OSCA from CCIs compared to those from family-based settings due to difficulties in following youth once they aged out of care. Thus, these results reflect the participants who remained in the OSCAR study long enough to complete at least one Phase 2 visit.

In conclusion, our results demonstrate that OSCA living in institutional environments are significantly more likely to complete primary school and attend secondary school but are somewhat less likely to complete secondary school than OSCA living in family-based care. For these children and youth who already face discrimination and very little to no support from their families, education may be especially important for future success. Further action is needed to reduce barriers to secondary school and improve completion rates among all OSCA. Policies forcing youth living in CCIs to leave upon turning 18 may pose a significant barrier to secondary school completion. Lessons from other countries demonstrate that OSCA continue to need support after turning 18. Efforts to improve care for OSCA in Kenya must build on strengths in the systems that currently exist and avoid replicating limitations seen in other jurisdictions.

## Data Availability

The datasets analysed during this study are not publicly available to protect individual privacy but are available from the corresponding author on reasonable request.
